# Does Metformin affect ER, PR, IGF-1R, β-catenin and PAX-2 expression in women with diabetes mellitus and endometrial cancer?

**DOI:** 10.1186/1758-5996-5-76

**Published:** 2013-12-05

**Authors:** Anna Markowska, Monika Pawałowska, Violetta Filas, Konstanty Korski, Marian Gryboś, Stefan Sajdak, Anita Olejek, Wiesława Bednarek, Beata Śpiewankiewicz, Jolanta Lubin, Janina Markowska

**Affiliations:** 1 Perinatology and Gynecology Department, Poznań University of Medical Sciences, Poznań, Poland; 2 Gynecological Oncology Department, Poznań University of Medical Sciences, Szamarzewskiego 82/84, 60-569 Poznań, Poland; 3 Department of Pathology, Poznań University of Medical Sciences, Poznań, Poland; 4 Wrocław University of Medical Sciences, Wrocław, Poland; 5 Clinic of Gynecological Surgery, Poznań University of Medical Sciences, Poznań, Poland; 6 Department of Gynecology, Obstetrics and Gynecological Oncology, Silesian Medical University, Bytom, Poland; 7 I Chair and Department of Oncological Gynecology and Gynecology, Lublin University of Medical Sciences, Lublin, Poland; 8 Department of Gynecology Oncology, Memorial Cancer Centre and Institute of Oncology, Warszawa, Poland

**Keywords:** Endometrial cancer, Metformin, Diabetes, PR, ER, IGF-1, IGF-1R, Beta catenin, PAX-2

## Abstract

**Objective:**

Diabetes mellitus, as a risk factor for endometrial cancer (EC), causes an increase in insulin and IGF-1 concentrations in the blood serum. The increase in insulin and IGF-1 are considered mitogenic factors contributory to cancer development. Studies suggest that metformin has preventive activity, decreasing mortality and the risk of neoplasms. Since estrogen (ER), progesterone (PR) and IGF-1 (IGF-1R) receptor expression and β-catenin and PAX-2 mutations are significant in the development of endometrial cancer, it was decided to study these factors in patients with endometrial cancer and type 2 diabetes mellitus (DM2), and to establish the effects of metformin on their expression.

**Methods:**

The expression of ER, PR, IGF-1R, β-catenin and PAX-2 have been immunohistochemically investigated in 86 type I endometrial cancer specimens. Patients were grouped according to the presence of DM2 and the type of hypoglycemic treatment administered.

**Results:**

Comparing EC patients with DM2 and normal glycemic status, we found increased IGF-1R expression in women with DM2. A decrease in ER expression was noted in women with EC and DM2 receiving metformin as compared to women treated with insulin (p = 0.004). There was no statistically significant difference in PR, IGF-1R, β-catenin and PAX-2 expression among women receiving metformin and other hypoglycemic treatment.

**Conclusion:**

Although epidemiological studies suggest the beneficial role of metformin in many human cancers, there are still few studies confirming its favorable effect on endometrial cancer. Decreased ER expression in patients receiving metformin needs further research to allow evaluation of its clinical significance.

## Background

Endometrial carcinoma (EC) is the sixth most commonly diagnosed cancer in women [1]. Risk factors for developing EC type 1, according to Bokhman division, are: imbalanced estrogen-progesterone ratios, advanced age, nulliparity, tamoxifen therapy, diabetes mellitus and obesity.

Among the women diagnosed with endometrial carcinoma, only 20-30% present normal body weight. Diabetes mellitus and surplus fat tissue are conducive to excessive androgen production and its peripheral conversion to estrone via aromatase. This mechanism is enhanced by hyperinsulinemia and IGF-1 overproduction, resulting in PI3K/Akt and MAPK pathway activation and consequently in increased cell division. An increased concentration of IGF-1 is linked to a decrease in sex hormone binding globulin (SHBG) production in the liver. Under normal circumstances, SHBG binds estrogens lowering their serum free fractional concentration, whereas decreased SHBG production increases the number of active hormones. Estrogen, not balanced by gestagen activity, stimulates endometrial cell proliferation, e.g. via local IGF-1 production, leading to endometrial hyperplasia and eventually to EC [2-4].

Metformin, a member of the biguanide family, is a widely used antidiabetic drug. Population studies show that metformin significantly reduces cancer risk in diabetic patients. A comparison of metformin patients with those taking sulfonylurea derivatives shows a 23% decrease in incidence of malignant neoplasms [5]. Metformin also lowers the risk of death from cancer. In a five-year observation period of 10,309 DM2 patients, a significant reduction in cancer mortality was noted among those on metformin when compared to patients on sulfonylurea derivatives or insulin (3.5% vs 4.9% vs 5.8%) [6], - esophageal, liver, colorectal, pancreatic, breast and lung cancers having the greatest risk reduction [7,8]. Recent studies have showed that metformin can inhibit cell proliferation and induce apoptosis in endometrial cancer cell lines [9].

The mechanism of metformin action is complex, all processes ultimately lead to enhanced tissue insulin sensitivity and reductions in blood glucose and insulin levels. The basic mechanism of metformin is to activate serine-threonine kinase (AMPK), a key protein in sustaining proper cell energy management. Metformin activates AMPK indirectly via a suppressor protein, liver kinase B1 (LKB1), and via the activation of tuberous sclerosis complex 2 (TSC-2) which inhibits the mammalian target of rapamycin (mTOR) protein, one of the key proteins in regulating cell division, protein synthesis, growth and angiogenic processes [10,11].

The mTOR protein is activated via the PI3K/Act pathway induced by insulin and growth factors, i.e.: IGF-1, EGF, PDGF and VEGF. High levels of insulin and IGFs in patients with DM2 and EC are contributive to mTOR overexpression, increased cell proliferation and resistance to apoptosis [12].

Additionally to the established role of estrogen and progesterone in hyperplasia induction and endometrial cancer onset, are other factors also involved in the development of this cancer, which include IGF-1R, β-catenin and PAX-2.

IGF-1 is a polypeptide produced in the liver similar in structure and function to insulin. After binding to its receptor (IGF-1R), signaling may occur through different mediators, the dominant pathway being PI3K/Akt, but also the MAPK (mitogen activated protein kinase) pathway. In the uterus, IGF-1 expression is strictly regulated by estrogen. Its signaling system is essential for cell differentiation, proliferation and migration. IGF-1 overexpression leads to neoplastic transformation, cancer progression and metastasis [13,14]. While examining the expression of IGF-1R in 152 cancers of various sites of origin, Ouban et al. [13] demonstrated high receptor membrane expression in breast cancer with prevalence of 87.5%, and of the ovary and endometrium with prevalence of 100%.

β-Catenin with E-cadherin play a role in preserving proper tissue architecture through the regulation of intercellular adhesion. Moreover, it constitutes part of the Wnt pathway that participates in the control of the expression of genes responsible for the normal course of the cell cycle, as well as for proliferation and for apoptosis. Mutations leading to the Wnt pathways excessive activation, are found in many malignant neoplasms including EC. Numerous studies show that β-catenin mutations may be crucial for carcinogenesis [15,16]. Because the studies evaluating β-catenin expression in the presence of DM2 are limited, we have decided to investigate if DM2 and its method of treatment change the role of β-catenin in EC.

The *PAX-2* gene encodes the transcript proteins involved in cell proliferation, differentiation and apoptosis. Mutations in these genes may result in modulation of the respective genes, thus contributing to oncogenesis [17,18]. A relationship between lowered *PAX-2* expression and ovarian cancer, endocervical adenocarcinoma and EC was described [19,20]. Recently, two *PAX-2* isoforms (*PAX-2A* and *PAX-2B*) have been discovered in pancreatic islets of Langerhans cells, one of their roles is to activate the glucagon gene expression responsible for the production of this hormone [21].

Since estrogen, progesterone and IGF-1 receptor expression is significant in EC, and mutations in β-catenin and *PAX-2* genes seems to be crucial in the neoplastic transformation of the endometrium, it was decided to study these factors in women with combined EC and DM2, and to determine a preventive effect of metformin on their expression.

## Methods

The study is multicentric and of retrospective character. The material consists of 150 archived samples of postmenopausal woman with type I endometrial cancer (endometrioid type) operated between 2007 and 2012. Patients with previous chemotherapy or radiotherapy were excluded.

All the samples were re-examined by H&E staining, from which 86 were stained immunohistochemically (IHC) (24 samples were excluded due to insufficient cancerous material, 15 – advanced autolysis, 2- premenopausal patients, 8- serous/clear cell carcinoma, 15- lack of proper documentation).

EC patients were divided in two groups according to the presence of DM2 – 48 DM2 subjects and 38 non-diabetic subjects (control group). Among the patients with DM2, 32 were treated with metformin in polytherapy (n = 10) or monotherapy (n = 22), the other 16 patients used insulin or sulfonylurea derivatives. Due to small number of patients receiving sulfonylurea derivatives in monotherapy (n = 6) we analyzed this subgroup together with patients treated with insulin (I + SD). Patient characteristics and the type of hypoglycemic drug used are shown in Tables 1 and 2.

**Table 1:** Patient characteristics (FIGO stage, grading) and type of hypoglycemic drug

**Endometrial cancer (n = 86)**					
	**Metformin in monotherapy**	**Metformin + other drugs**	**Insulin in monotherapy**	**Sulfonylurea derivatives**	**Non- diabetics (control)**
	**(n = 22)**	**(n = 10)**	**(n = 10)**	**(n = 6)**	**(n = 38)**
FIGO I	19	9	8	6	32
FIGO II-IV	3	1	2	0	6
G1	8	6	6	3	23
G2	13	3	3	3	11
G3	1	1	1	0	4

**Table 2:** Detailed patient characteristics (age, FIGO stage, grading) and type of hypoglycemic drug

**No**	**D.t2**	**Drug**	**Age**	**FIGO**	**Grading**	**No**	**D.t2**	**Age**	**FIGO**	**Grading**
**1**	+	Met	60	IA	G2	**1**	-	58	IA	G1
**2**	+	Met	61	IB	G1	**2**	-	61	IB	G2
**3**	+	Met	57	IA	G1	**3**	-	43	IA	G1
**4**	+	Met	63	IA	G1	**4**	-	73	IA	G1
**5**	+	Met	73	IA	G2	**5**	-	59	II	G2
**6**	+	Met	62	IB	G1	**6**	-	63	IA	G1
**7**	+	Met	63	II	G2	**7**	-	56	IA	G1
**8**	+	Met	74	IB	G2	**8**	-	59	IA	G2
**9**	+	Met	74	IA	G1	**9**	-	52	IA	G1
**10**	+	Met	73	IA	G1	**10**	-	61	IA	G1
**11**	+	Met	69	IB	G2	**11**	-	55	IB	G1
**12**	+	Met	60	IA	G2	**12**	-	63	IB	G2
**13**	+	Met	71	II	G2	**13**	-	70	IA	G2
**14**	+	Met	68	IA	G2	**14**	-	62	IA	G1
**15**	+	Met	72	IB	G1	**15**	-	54	IA	G1
**16**	+	Met	58	IB	G2	**16**	-	56	IA	G1
**17**	+	Met	80	II	G2	**17**	-	76	IIIA	G3
**18**	+	Met	57	IB	G3	**18**	-	65	II	G2
**19**	+	Met	71	IA	G1	**19**	-	52	IA	G1
**20**	+	Met	78	IB	G2	**20**	-	69	IA	G1
**21**	+	Met	65	IA	G2	**21**	-	71	IA	G2
**22**	+	Met	76	IA	G2	**22**	-	80	II	G1
**23**	+	Met + Ins	61	IB	G3	**23**	-	67	IA	G2
**24**	+	Met + Ins	54	IA	G1	**24**	-	58	IV	G2
**25**	+	Met + Ins	80	IA	G1	**25**	-	56	IIIA	G3
**26**	+	Met + Ins	72	IA	G2	**26**	-	71	IB	G2
**27**	+	Met + Ins	73	IB	G1	**27**	-	59	IB	G1
**28**	+	Met + Ins	71	IVB	G1	**28**	-	71	IB	G1
**29**	+	Met + Ins	64	IA	G2	**29**	-	59	IB	G3
**30**	+	Met + Ins	69	IA	G2	**30**	-	68	IA	G1
**31**	+	Met + Ins	61	IA	G1	**31**	-	65	IB	G1
**32**	+	Met + Sulf	58	IA	G1	**32**	-	71	IB	G2
**33**	+	Ins	68	IB	G1	**33**	-	66	IB	G1
**34**	+	Ins	66	IA	G1	**34**	-	59	IB	G3
**35**	+	Ins	74	IIIB	G3	**35**	-	76	IB	G1
**36**	+	Ins	76	IB	G2	**36**	-	52	IA	G1
**37**	+	Ins	72	II	G1	**37**	-	76	IB	G1
**38**	+	Ins	69	IA	G2	**38**	-	54	IB	G1
**39**	+	Ins	73	IA	G2					
**40**	+	Ins	61	IA	G1					
**41**	+	Ins	72	IA	G1					
**42**	+	Ins	63	IB	G1					
**43**	+	Sulf	55	IA	G2					
**44**	+	Sulf	63	IA	G1					
**45**	+	Sulf	50	IB	G1					
**46**	+	Sulf	72	IB	G2					
**47**	+	Sulf	61	IA	G1					
**48**	+	Sulf	83	IB	G2					

D t.2- diabetes type 2; Met- metformin; Ins- insulin; Sulf- sulfonylurea derivatives.

This study was approved by the Ethics Committee of the Poznan University of Medical Sciences.

The average age of the patient with EC was 65.25. Patients with EC and DM2 were older than the EC control group (67.2 vs 62.8 p = 0.01). The average BMI in all of the EC patients was 32.1 kg/m2. Patients with EC and DM2 had a higher BMI index than EC patients without diabetes (33.6 vs 30.5 p = 0.01). In relation to the methods of anti-diabetic treatment, there was no difference in the average age and BMI of DM2 cancer patients.

In order to assess the relationship between protein expression and the FIGO stage of EC, the patients were divided into two subgroups- FIGO I and FIGO II-IV stages.

A total of 86 preparations were IHC stained to determine the presence of ER (clone SP1), PR (clone 636), IGF-1R (clone G11) (Ventana Medical Systems, Inc), β-catenin (clone 14) (Dako Denmark) and PAX-2 (clone 3C7) (AbD Serotec). IHC analysis was performed using the UltraView DAB Detection Kit system by Roche Group. Immunoperoxidase staining was performed using the Ventana BenchMark Ultra.

The nuclear receptor expression was assessed by counting point values (SCORE) equal to the sum of average intensity of positive-staining cancer cells (scale from 0 to 3, where 0 stands for no, 1- weak, 2- intermediate, 3- strong intensity of staining) and the percentage of stained cells (scale from 0 to 5; 0- none; 1- < 1%; 2- 1%-10%; 3- 10%-33.3%; 4–33.3%-66.6% and 5- >66.6%) [22]. For proteins that displayed a membranous reaction, only the percentage of positively stained cells (from weak to strong intensity of staining) in the cancer tissue were determined (FIELD).

The assessment was performed by two separate investigators in blinded fashion, each person scored the staining in ten HPFs (high power fields). Due to a substantial number of samples stained, the batch analysis was carried out by comparison of staining intensity of positive controls used in every batch.

In statistical calculations, Mann-Whitney’s and Spearman’s tests were used (STATISTICA, StatSoft Inc, USA). Statistical significance was set at a p value < 0.05.

## Results

**Figure 1:**
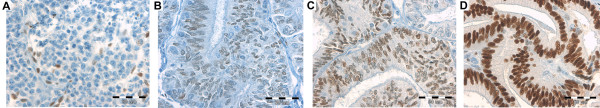
**Different intensities of nuclear immunostaining for ER. A**- no; **B**- weak; **C**- intermediate, **D**- strong intensity of staining.

**Figure 2:**
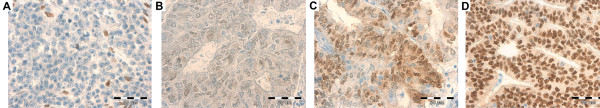
**Different intensities of nuclear immunostaining for PR. A**- no; **B**- weak; **C**- intermediate, **D**- strong intensity of staining.

**Figure 3:**
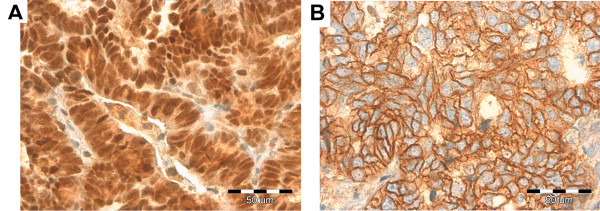
Nuclear immunostaining for nuclear PAX-2 (A) and IGF-1R membranous type of staining (B).

**Figure 4:**
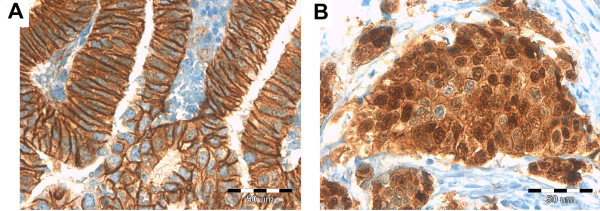
Membranous (A) and nuclear (B) type of immunostaining for β-catenin.

Table 3 represents mean percentage values of IHC positively stained cells for ER, PR, PAX-2, IGF-1R and β-catenin in studied groups. ER, PR and PAX-2 demonstrated a nuclear type of reaction (Figures 1, 2, 3). IGF-1R staining was of a membranous type (Figure 3). β-catenin demonstrated two staining patterns: one being nuclear and the other a dominant, membranous reaction (Figure 4).

**Table 3:** Mean percentage (± SD) of positive immunohistochemically stained cells (IGF-1R and β-catenin) and mean SCORE results (ER, PR and PAX-2) in patient subgroups

	**PR score**	**ER score**	**PAX-2 score**	**IGF-1R**	**β-catenin (membranous)**
**EC + diabetes (n = 48)**	6.85 ± 1.76	6.91 ± 1.51	3.58 ± 2.42	36.79 ± 26.73	86.87 ± 19.17
**M in mono- and polytherapy (n = 32)**	6.78 ± 2.01	6.71 ± 1.67	3.37 ± 2.41	36.59 ± 24.49	83.75 ± 21.28
**M in monotherapy (n = 22)**	6.86 ± 1.83	6.77 ± 1.15	3.5 ± 2.64	36.41 ± 26.16	84.54 ± 20.23
**I + SD (n = 16)**	7.0 ± 1.15	7.31 ± 1.07	4.37 ± 2.02	37.18 ± 31.72	93.12 ± 12.36
**I in monotherapy (n = 10)**	7.1 ± 1.28	7.8 ± 0.42	4.2 ± 2.34	33.5 ± 28.96	97.0 ± 6.32
**Control (n = 38)**	6.39 ± 2.23	6.28 ± 2.17	3.34 ± 2.31	18.29 ± 27.27	87.5 ± 24.40

EC- endometrial cancer, M- metformin, I- insulin, SD- sulfonylurea derivatives, I + SD- Due to small number of patients receiving sulfonylurea derivatives in monotherapy (n = 6) we have analyzed this subgroup together with patients treated with insulin.

### ER

In our material, although we observed decreasing expression of ER parallel with decreasing histological differentiation of cancer cells, we did not find statistically significant difference in ER expression according to the grade of EC (G1 vs G2 p = 0.99; G2 vs G3 p = 0.14; G1 vs G3 p = 0.13). No correlation with FIGO staging was observed. No difference in the positive ER rate was found when comparing diabetic and non-diabetic subgroups of the EC patients (p = 0.18). However, it was shown that DM2 women with EC receiving insulin in monotherapy had a significantly higher ER expression than non-diabetic women with EC (p = 0.0046).

**Figure 5:**
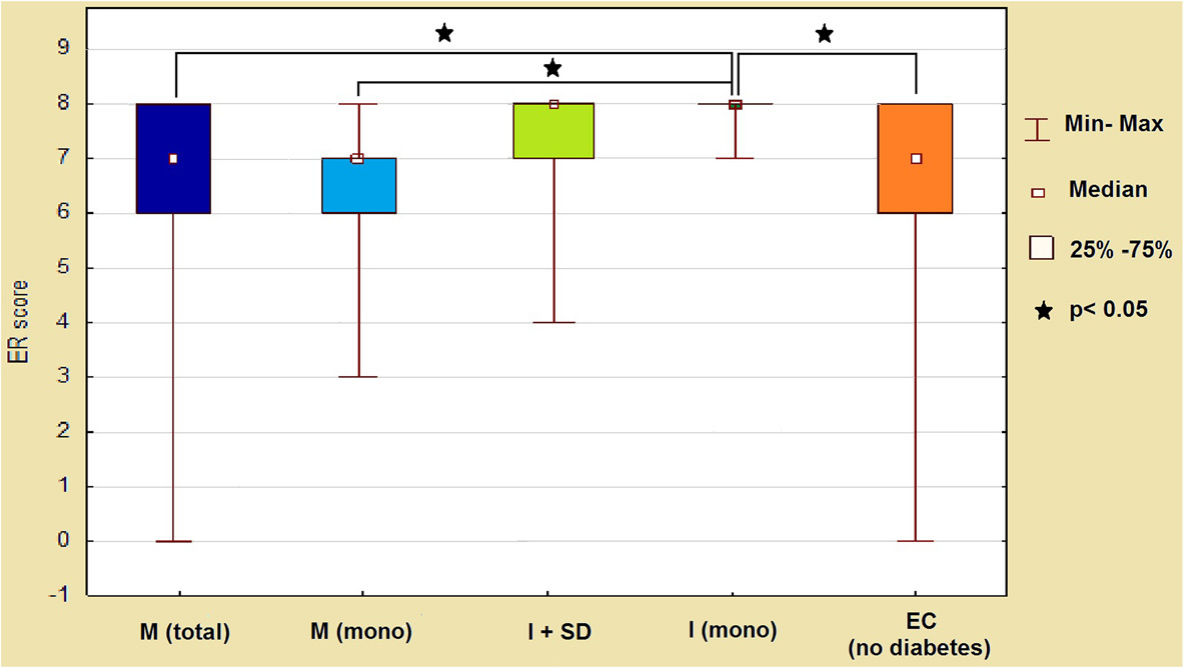
**ER expression (score) in endometrial cancer cells according to presence of diabetes and method of its treatment.** M- metformin, I- insulin, SD- sulfonylurea derivatives.

Moreover, patients treated with metformin demonstrated a statistically significant reduction in ER expression in comparison to the group receiving insulin in monotherapy (p = 0.014 for all patients treated with metformin; p = 0.004 for metformin in monotherapy) (Figure 5).

### PR

Comparing EC of low and high grade, a reduced PR expression was found in samples with poorly differentiated cells (G1 vs G3 p = 0.029; G1 vs G2 and G2 vs G3 p > 0.05). However, no differences were observed in PR expression in relation to FIGO staging, the presence of diabetes, and the method of its treatment (p > 0.05).

### IGF-1R

No difference in the rate of positively stained cells was found in the EC group in relation to grading and FIGO staging.

Cancer patients with diabetes showed a significantly higher IGF-1R expression then the non-diabetic group (p = 0.0012). This trend is present in groups of women treated with metformin (p = 0.0056) or other drugs (p = 0.01). Comparing different types of anti-diabetic treatments in EC patients, we did not find any significant changes in the expression of IGF-1R (p > 0.05).

### β-Catenin

All cases under study, excluding one (a case of FIGO IIIA G3 endometrioid cancer in a non-diabetic patient with a strong nuclear reaction) demonstrated a dominant, membranous reaction of β-catenin.

The group of diabetic EC patients did not show a difference in membranous protein expression in comparison to the control groups (p = 0.32). No differences were demonstrated in relation to cancer grading or clinical advancement of the disease according to FIGO (p > 0.05). However, although not statistically significant, patients on metformin monotherapy displayed a lower protein expression than those on insulin monotherapy (p = 0.07).

A nuclear reaction was found in 12 out of 86 EC samples (13.9%), 8 cases concerning non-diabetic and 4 cases concerning diabetic patients (8.3% and 21.5%, respectively for each group). Among diabetic patients, two cases were treated with metformin and two with insulin or sulfonylurea derivatives. Positive nuclear staining was found at both early and advanced stages of the disease and with low and high grade differentiation (Table 4).

**Table 4:** Nuclear expression of β-catenin according to the presence of endometrial cancer, diabetes and method of its treatment

	**EC - Total**	**EC DM2 – Metformin**	**EC DM2 - Insulin or sulfonylurea derivatives**	**EC – Control group**
	**(n = 86)**	**(n = 32)**	**(n = 16)**	**(n = 38)**
**B-CATENIN**	12 (13,9%)	2 (8,3%)	2 (12,5%)	8 (21,5%)
**FIGO**	IA- 4	IA- 1	IA- 0	IA- 3
	IB- 5	IB- 1	IB- 2	IB- 2
	II- 1	II- 0	II- 0	II- 1
	IIIA- 2	IIIA- 0	IIIA- 0	IIIA- 2
**GRADING**	G1- 4	G1- 1	G1- 1	G1- 2
	G2- 5	G2- 0	G2- 1	G2- 4
	G3- 3	G3- 1	G3- 0	G3- 2

### PAX-2

The intensity of the nuclear reaction for PAX-2 was not statistically varied between DM2 EC patients and control groups (p = 0.53). No differences were observed between FIGO staging and grading, and the type of anti-diabetic drug administered (p > 0.05).

## Discussion

### ER, PR, IGF-1R

The increased risk of developing EC in women with DM2 is an indisputable fact [23]. There is also clear evidence showing a positive association between the increased levels of circulating insulin and the incidence of endometrioid adenocarcinoma [24,25]. But despite numerous studies, there is still no unanimous data explaining the role of IGF-1 on endometrial carcinogenesis. Additionally, there is also a lack of consensus on the prevailing type of receptor (IR or IGF-1R) acting on EC promotion.

Endometrial cancer type I is a hormone dependent malignant disease, in which the balance between estrogen and progesterone is disrupted and an increase of estrogen stimulation leads to excessive cell proliferation.

High ER and PR expression in EC are frequently connected with endometrioid type of neoplasm, better cells differentiation, lower risk of lymph node metastases and better prognosis [26,27]. Based on studies of endometrial and breast cancer tissue, we can observe a profound and complex crosslink between estrogen, progesterone, insulin or IGF-1 and its receptors.

In the normal endometrium, especially during the proliferative phase, estrogen binding to ER receptor acts as a transcription factor triggering local IGF-1 production [28]. On the other hand, endometrial stromal cells, due to progesterone induction, produce IGFBP-1 (IGF-1 binding protein), which after binding to IGF-1 reduces its bioavailable fraction. Mitogenic function of IGF-1 may occur through different mediators, the dominant pathway being PI3K/Akt, but also MAPK, both involved in regulation of cell proliferation and apoptosis. Furthermore, active MAPK signaling can phosphorylate serine in NH2-terminal region of the ER- AF1, and thereby increase ER activation [29]. Estrogen not only influences IGF-1 production but also takes part in the regulation of IGF-1R, elevating its endometrial expression [30]. The inhibition of IGF-1R promotor may occur via e.g. p53 [31], but its mutation is often seen in EC leading to IGF-1R overexpression.

High IGF-1 plasma concentrations and the presence of IGF-1R on the cell surface are found in many cancers [32,33], but in the case of EC, the role of circulating IGF-1 is controversial. It was noted that high fasting glucose levels in women not on hormone therapy was correlated with the development of endometrioid adenocarcinoma, whereas the rise in free IGF-1 plasma fraction, has no effect or even decreases the risk of developing EC [24,34]. These observations may suggest the dominant role of the local production of IGF-1 in the neoplastic endometrium. Most likely, the rise of local free IGF-1 in diabetic patients is due to insulin mediated inhibition of the production of IGFBP-1 [35].

In EC tissue, IGF-1 can influence PR expression. Its high levels are associated with improved prognosis and response to gestagen treatment, especially in advanced or recurrent carcinoma [36]. Xie et al. [37] observed IGF-1 and IGF-2-induced reduction of PR in EC cell lines, which was related to the activation of the PI3K/Akt/mTOR pathway and phosphorylation of the p70S6K effector protein. In an in vitro study, metformin was found to inhibit the growth of ECC-1 and Ishikawa EC cells in a dose dependent manner via activation of AMPK and inhibition of mTOR [9]. It was noted that the administration of this drug can raise PR expression in EC [37]. Similar results were presented by Berstein et al. [38] in 90 breast cancer samples from patients with DM2. In immunohistochemical assessment of ER and PR researchers found no difference in the ER expression of cancer cell in women receiving metformin, insulin, sulfonylurea derivatives and those who were exclusively on a diabetic diet. However, an increased percentage of positive PR in breast cancer specimens was found in patients treated with metformin mono- or polytherapy (p = 0.04).

Our study does not show any difference between PR expression in patients receiving different types of pharmacotherapy in DM2. However, we found a reduction in the rate in which cells displayed a strong ER reaction in EC patients receiving metformin in comparison to those patients on insulin monotherapy. It is believed that metformin may decrease estrogen concentration in neoplastic tissue via local inhibition of aromatase activity suppressing synthesis of the enzyme through interaction with its promoter, PII [39]. But the exact mechanism linking metformin uptake with ER reduction is unknown. We can speculate that the reduction of ER after metformin treatment may decrease the number of cells sensitive to high levels of estrogens, affecting their proliferative abilities and at the same time may influence the prognosis. But further studies are needed to confirm this hypothesis.

Pengchong H and Tao H. [40] showed a greater IGF-1R expression in EC than in normal endometrium and indicated a correlation between IGF-1R overexpression and metastasis to the lymph nodes. Roy et al. [41] in turn found no statistically significant differences between the amount of mRNA IGF-1R in normal cells and EC cells. Interestingly, there was a significant reduction in IGF-1 expression in EC when comparing the endometrium at proliferative (p = 0.0078) and early secretory (p = 0.0002) phases. This disproportion between a relatively high IGF-1R and low IGF-1 in EC cells may reflect intense disruptions in IGF-1R encoding gene expression, leading to protein overproduction (e.g. mutations in suppressor genes i.e. *p53* or *BRCA1* resulting in the lack of inhibitory effect on the IGF-1R promotor).

In our material women with EC and diabetes demonstrated a significantly higher IGF-1R expression in comparison to the non-diabetic women. This may be associated with e.g. different affinity to specific receptors (IR- α and β, IGF-1R, hybrid receptors) of insulin, and the circulating IGF-1 and IGF-2 or their local production in neoplastic tissue. Metformin administration did not significantly influence the number of IGF-1R in EC. Presumably, the drug inhibits further stages of the PI3K/Akt/mTOR pathway initiated by IGF-1R, without direct influence on IGF-1R membrane expression. In future research it is valid to compare the expression and activity not only of IGF-1R but also of IR isoforms in normal and neoplastic endometrium of diabetic and non-diabetic patients and to assess the influence of metformin on their expression.

### Beta-catenin

β-Catenin (*CTNNB1*) plays a double role in cells –first, it regulates cellular adhesion via interaction with membrane protein E-cadherin – second, in the cell nucleus it plays a key role in the Wnt signaling pathway.

The normal endometrium displays a strong membranous reaction of β-catenin. However, a reduction in β-catenin and E-cadherin expression is noted in different types of neoplastic tissue, including endometrial tissue. Seagusa et al. [42] revealed a gradual decrease in cell membrane β-catenin immunoreactivity from normal to atypical hyperplasia and to grade 3 carcinomas. Disruption in both β-catenin and E-cadherin expression is associated with reduced cell to cell adhesion, the advancement of the clinical stage of the disease and the increased risk of metastasis [15,16].

The Wnt signaling pathway is a multi-protein complex consisting of axins, β-catenin, APC (adenomatous polyposi coli), kinase CKI (casein kinase I) and GSK3β (glycogen synthase 3β). In the absence of Wnt ligands, the APC/GSK3β/Axin complex is activated which results in phosphorylation of β-catenin and its ubiquitination and proteasomal inactivation. Once the Wnt pathway is activated, APC/GSK3β/Axin does not form a complex with β-catenin and phosphorylation of this protein is inhibited. Stabilized β-catenin is translocated to the nucleus and is assembled into a complex with transcription factors LEF (lymphoid enhancer factor) and TCF (T-cell factor), promoting expression of many genes involving in cell proliferation (Myc or cyclin D1), angiogenesis (VEGF), adhesion and apoptosis (survivin).

Activation of β-catenin/Wnt pathway is a physiological phenomenon during the menstrual cycle, but it differs among cycle phases. Nei et al. [43] showed the presence of nuclear β-catenin in the proliferative phase, whereas during the secretory phase the protein was found mainly in the cytoplasm and the cell membrane.

Estradiol can change the expression of the Wnt ligands including Wnt 4 and 7A [43,44]. Furthermore, it is also linked to Wnt and PI3k/Akt signaling pathways. It was found that after estrogen stimulation, ER alpha induced PI3K and subsequently activated Akt. This in turn resulted in phosphorylation and inhibition of GSK3 [45]. Consequently, β-catenin cannot be phosphorylated and degraded in proteasomes.

A number of studies indicate disruptions in β-catenin expression in EC, most frequently in the endometrioid type. Mutations in *CTNNB1,* but also in other genes encoding proteins taking part in Wnt pathway (APC or axin), result in stabilization of β-catenin, its excessive nuclear accumulation and promotion of many genes which lead to neoplastic transformation [46]. Konopka et al. [47] found mutations in the *CTNNB1* region in 16.1% of ECs. The mutations detected at the atypical hyperplastic endometrium and the early stages of EC suggest their significant role in early carcinogenesis [44].

Limited literature concerning β-catenin expression in diabetic EC patients makes it impossible to compare our findings to other studies. In our material concerning EC, β-catenin nuclear staining was found in 13.9% of cases which corresponds accordingly to the Nout et al. research [48] of 14%. In our findings the majority of cases concerned non-diabetic patients with EC (21.5% vs 8.3% of patients with diabetes) (Table 3). Due to a small number of diabetic patients with a positive nuclear reaction, it is difficult to assess the influence of anti-diabetic treatment on nuclear accumulation of β-catenin. However, if metformin reduces the expression of ER, which was demonstrated in our study, it is presumed that it may also decrease the activation of PI3K/Akt signaling, increasing the unphosphorylated fraction of GSK3 and reducing the amount of β-catenin. Further studies are necessary to examine the correlation between β-catenin expression, Wnt pathway activation and diabetes in women with EC.

### PAX-2

PAX-2 participates in regulating the proper development of the central nervous system, the kidneys and the Müllerian ducts (upper part of the vagina, uterus, fallopian tubes). Even more evidence indicates that it also has a significant role in oncogenesis, including EC.

Monte et al. [20] described the loss of PTEN and PAX-2 expression in normal, hyperplastic, and cancer cells, indicating that independent from PTEN, PAX-2 acts as a suppressor gene undergoing inactivation during cancer transformation. In the normal endometrial tissue, versus precancerous lesions and cancer, the level of the PAX-2 protein loss increases progressively at the rate of 36%, 71%, and 77% respectively. Similar results were obtained by Allison et al. [49], which suggests that PAX-2 protein losses occur at an early stage of carcinogenesis. Unfortunately the mechanism explaining this phenomenon is yet unknown.

Researchers do not know of any study regarding PAX-2 expression in EC in relation to coexisting glucose tolerance disorders.

In our material, no difference in PAX-2 nuclear expression was found in patients with EC in relation to the presence of diabetes or the type of treatment administered. However, these results may be considered as questionable, because of the strong staining of the cytoplasm which may have deterred the assessment of the nuclear reaction. Further research is needed in order to determine if there is a relationship between PAX-2 expression and diabetes in patients with EC, if proven so, then to additionally determine the influence of metformin administration.

## Conclusion

Diabetes as a risk factor in EC increases insulin and IGF-1 blood levels, both considered mitogenic factors contributing to the development of many cancers via enhanced cell proliferation. Many trials as well as ongoing clinical research suggest a preventive effect of metformin, not only in the context of malignant neoplasms, but also as a factor for improved prognosis and reduced mortality among cancer patients. With the use of immunohistochemical assessment, this research found a reduction in ER expression in diabetic women receiving metformin. Such relationship was not found for PR. Although, enhanced IGF-1R expression has been observed in EC of diabetic patients, no statistically significant difference were found among patients receiving metformin, insulin or sulfonylurea derivatives. Additionally, no statistically significant differences have been noted in the β-catenin and PAX-2 reaction for any of the compared groups. Further research is necessary to assess the effects of the abovementioned proteins in the prognosis of patients with EC and diabetes treated with metformin.

### Consent

Written informed consent was obtained from the patient for the publication of this report and any accompanying images.

## Competing interests

The authors declare that they have no competing interests.

## Authors’ contributions

AM- author of the conception of the study; designed the study and completed scientific team. MP- carried out immunohistochemical assessment; participated in performing the statistical analysis and coordination of the project; drafted the manuscript. VF- carried out the immunoassays. KK^-^ carried out immunohistochemical assesment. MG- collection of data; participated in the design of the study. SS- collection of data; participated in the design of the study. AO- collection of data; participated in the design of the study. WB- collection of data; participated in the design of the study. BŚ- collection of data; participated in the design of the study. JL- participated in interpretation of data; participated in drafted the manuscript. JM- participated in the design of the study and coordination; interpretation of data. All authors read and approved the final manuscript.
